# Spatiotemporal dynamics of suspended sediment in coastal Mekong Delta: a hydrodynamic modelling approach under tropical monsoon climate

**DOI:** 10.1038/s41598-025-89111-z

**Published:** 2025-02-18

**Authors:** Nguyen Ngoc An, Pham Viet Hong, Nguyen An Binh, Giang Thi Phuong Thao, Le Van Tinh, Nguyen Cao Hanh, Thai Thanh Tran

**Affiliations:** 1 Ho Chi Minh City Institute of Resources Geography, Vietnam Academy of Science and Technology, Ho Chi Minh City, Vietnam; 2 Institute of Marine Geology and Geophysics, Vietnam Academy of Science and Technology, Hanoi, Vietnam; 3 Ho Chi Minh City University of Natural Resources and Environment, Ho Chi Minh City, Vietnam; 4 Institute of Tropical Biology, Vietnam Academy of Science and Technology, Ho Chi Minh City, Vietnam

**Keywords:** Environmental sciences, Hydrology

## Abstract

Suspended sediment concentration (SSC) plays a pivotal role in shaping coastal dynamics, impacting terrestrial and marine ecosystems. This study employed the hydrodynamic model MIKE21 to simulate hydrological runoff and sediment transport within the Mekong River’s fluvial-marine continuum, the longest river in Southeast Asia currently challenged with escalating anthropogenic pressures and sea-level rise. By strategically selecting hourly observed data from various locations (river channel, coastal estuary) and periods (dry and rainy seasons) for model calibration and validation, we demonstrated the robust performance of the model simulation of both water levels (RMSE: 0.343 m) and SSC (RMSE: 0.006 kg.m^−3^). Spatiotemporal analysis of 2019–2023 revealed the pronounced sensitivity of water level, velocity, and flow direction under tropical monsoon regime. SSC time series decomposition further extracted seasonal amplitudes, while spatial patterns showed distinctly the lowest concentrations occurring in April and the highest in September annually. Furthermore, SSC upward trends were observed during low-flow periods, while downward trends predominated during high-flow periods. Our quantitative analysis offers a comprehensive understanding of hydrological processes within tropical monsoon coastal regions. These findings support the establishment of long-term monitoring frameworks to inform nature-based strategies for sustainable coastal development.

## Introduction

Coastal deltas, situated at the confluence of marine and terrestrial biospheres, represent one of the most complex environmental systems worldwide. Characterized by diverse ecosystems, including estuaries, mangrove forests, seagrass meadows, coral reefs, salt marshes, sandy beaches, and rocky shores, these areas stand out as hot spots of biodiversity with unique ecological functions^1^. In terms of supporting sustainable development goals (SDG) outlined by the United Nations, coastal regions play an indispensable role in achieving various targets, particularly in the conservation of both terrestrial (SDG 14) and marine ecosystems (SDG 15)^2^. Compared to the early periods of twenty-first century, coastal deltas are anticipated to experience rapid urbanization rates in the coming decades^3^. Consequently, these terrestrial-marine environments are increasingly vulnerable to the combined pressures of climate change, sea-level rise, and anthropogenic activities. A recent report highlighted the disproportionate impact of infrastructure-mediated coastal squeeze in Asia^4^, while Vietnamese coastal regions are expected to rank top ten counties with the highest exposure to natural hazards in the future^2^.

The Mekong River, a Southeast Asian waterway spanning approximately 4,900 kilometers^5^, ranks as the longest river in Southeast Asia^6^ and the seventh-largest river globally^7^. It plays a pivotal role in supporting productive fisheries, providing abundant freshwater for agriculture and mitigating soil salinity, and serving as a significant source of sediment for the formation of alluvial floodplains and coastal regions in the countries it traverses (i.e., 80 million tons/year)^8^. Notably, the Mekong River’s sediment load is comparable to that of the Mississippi River, constituting 85% of the Yangtze River’s sediment load and surpassing the Amazon River by 12%^9^. The river originates in the Tibetan Plateau and flows through Vietnam, bifurcating into the Tien River and the Hau River. With a total flow of approximately 2300 m^3^/s during the dry season and 40,000 m^3^/s during the rainy season^10,11^, the river ultimately discharges into the East Sea through nine estuarine entrances, collectively known as the Cuu Long River. The Hau River’s distributary accounts for approximately 41% of the total water volume entering the coastal waters through the Dinh An and Tran De estuaries^12^.

Suspended sediment plays a pivotal role in shaping aquatic ecosystems, providing substantial alluvium to deltas and essential nutrients to coastal environments^13,14^. The ecology and morphology of rivers, estuaries, and marine habitats are significantly influenced by the presence and characteristics of suspended sediments^15–17^. Variations in sediment flux and particle size can have profound consequences for nutrient availability, downstream erosion, reservoir sedimentation, and pollutant distribution^18,19^. Excessive sediment loading can adversely affect delicate ecosystems like coral reefs^20^. Furthermore, sediment possesses the capacity to sequester pollutants from aquatic environments and store them at the basin floor^21–23^, while also serving as carbon sinks^24,25^. This dual function contributes significantly to mitigating global warming and remediating river pollution.

As growing concerns regarding sediment regime, recent advancements have witnessed a paradigm shift from traditional geological sampling methods to hydrodynamic modelling, facilitating the monitoring of spatiotemporal variations across regional, continental, and global scales^26,27^. Hydrodynamic modelling also allows for the exploration of the intricate interplay between physical processes and suspended sediment concentration (SSC) dynamics under the influence of both anthropogenic activities and natural conditions. For instance, two-dimensional hydrodynamic models have been employed to simulate the origin, particle size, transport, and distribution of SSC before and after reservoir construction and dam building, as well as to evaluate the changes in river depth and sediment deposition both within and beyond the reservoir^28–30^. Integrating the hydrodynamic model with sea-level rise scenarios enables the assessment of the reduction in SSC attributable to future wave and tidal height variations^31,32^.

In the wake of escalating challenges posed by climate change, coastal urbanization, hydropower dam construction, and water discharge within the Mekong Delta, the temporal and spatial dynamics of sediment transport and distribution have become increasingly critical to monitor. This study comprehensively investigates SSC and distribution within the Lower Mekong Delta, focusing on the Hau River over the latest period from 2019 to 2023. Utilizing a numerical modelling approach, we simulated the spatiotemporal dynamics of water level, flow velocity, direction, and SSC under the influence of the tropical monsoon regime. Time series decomposition revealed subsequently pronounced seasonal variations accompanied by precise trends. Our findings provide valuable insights into model selection and configuration, as well as quantifying seasonal amplitude and long-term trend over the river-estuarine continuum with significant annual low–high flow oscillations driven by the region’s tropical monsoon climate.

## Results

### Performance of hydrological simulation

**Fig. 1:**
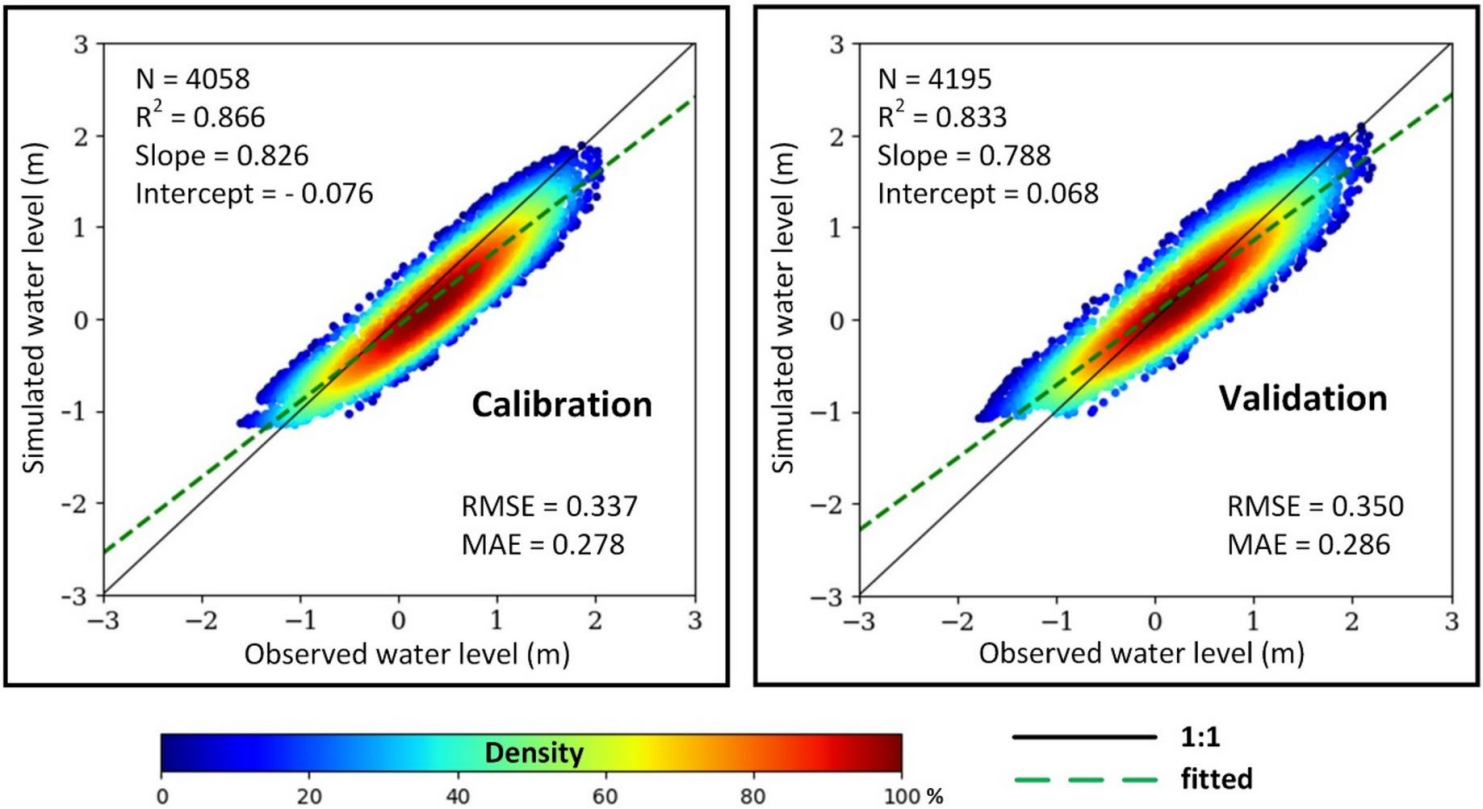
Performance of water level simulation in calibration (left) and validation (right). All observed data collected by Tran De (TD) and Dai Ngai (DN) stations over 2019, among three dry months (February, March, and April) for calibration and three rainy months (August, September, and October) for validation.

Figure 1 presented the performance of the water level simulation model following calibration and validation (see Supplementary Figures S1 and S2 for comparison in monthly time series). The model exhibited satisfactory performance, as determined by the correlation coefficients (R^2^) exceeding 0.8. The slope values ranging from 0.788 to 0.826 suggest a slight underestimation of water levels. A rigorous evaluation against over 4000 hourly station observations confirmed the robustness of the model, with RMSE and MAE determined to be 0.343 m and 0.282 m in calibration and validation, respectively. Notably, the model effectively captured the pronounced variations in water level during both dry and rainy periods when employing distinct calibration and validation datasets, highlighting its adaptability to diverse hydrological conditions within the study area.

**Fig. 2:**
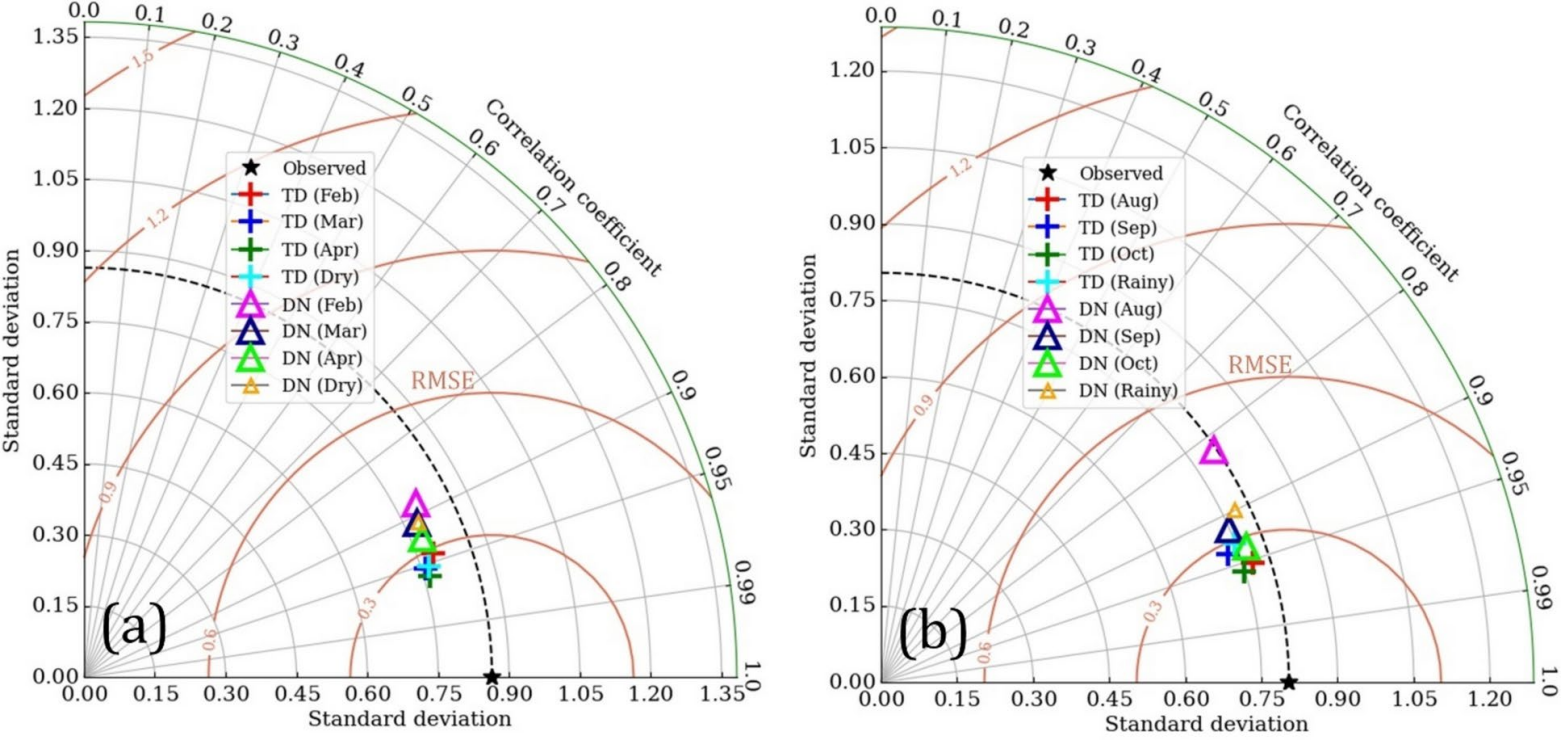
Taylor diagram summarized the performance of water level simulation separated by individual station and period: dry season represents calibration (left), and rainy season represents validation (right). The black dotted line represents the average standard deviation of the water level observed in stations.

Multi-metric Taylor diagrams (Fig. 2) illustrated water level simulation efficiency under complex runoff conditions from inland (DN station) to estuarine (TD station) environments, encompassing both seasons and individual months. Visual comparisons clearly demonstrated superior model performance at the TD station relative to the DN station for both calibration and validation phases. For instance, the DN station exhibited peak errors in February (RMSE = 0.4 m, R = 0.88) and August (RMSE = 0.49 m, R = 0.82), while all TD station RMSE values remained below 0.3 m across all evaluations. The complex hydrodynamics of the narrow river channel, compounded by agricultural water management, render the inland water environment more challenging to model. Nevertheless, obtained error values fall within acceptable limits, and the negligible differences in performance metrics between the two stations support the model’s overall reliability.

**Fig. 3:**
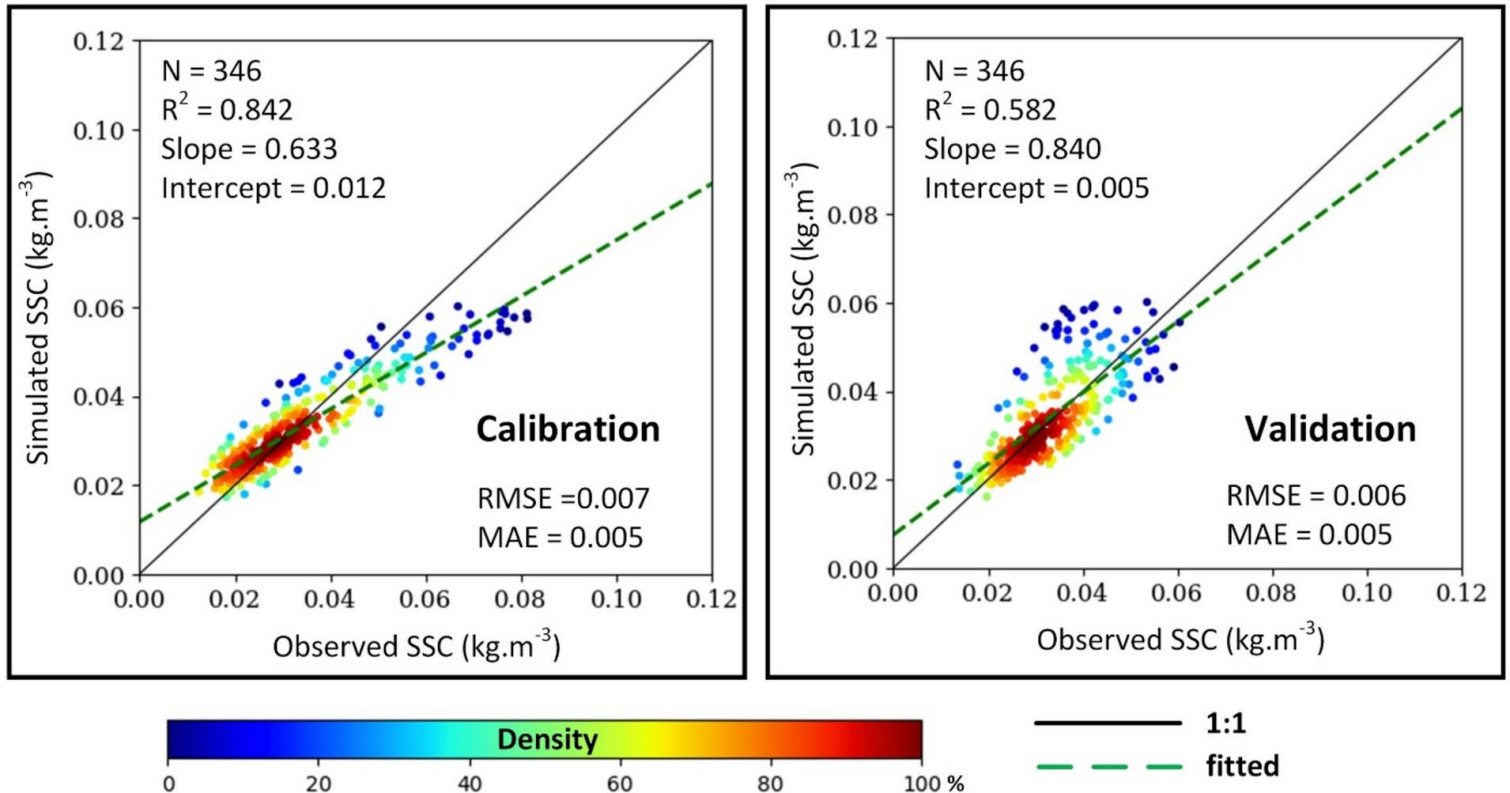
Performance of SSC estimation based on data collected in Can Tho (CT) stations in 2019 and 2021 used for calibration (left) and validation (right), respectively.

Transitioning to the sediment transport, a moderate decrease in correlation coefficient was observed between the calibrated (R^2^ = 0.84) and validated (R^2^ = 0.58) process (Fig. 3). However, an increase in the slope of the relationship between observed and simulated SSC in validation (0.84) indicated the fitness following calibration. Additionally, RMSE decreased by 0.001 kg m^−3^ and MAE (0.005 kg m^−3^) remained consistent in the validated model, suggesting reliable model simulations (see Supplementary Figures S3 and S4 for comparison in time series).

### Fluid dynamics and sediments transport over space and time dimensions

**Fig. 4:**
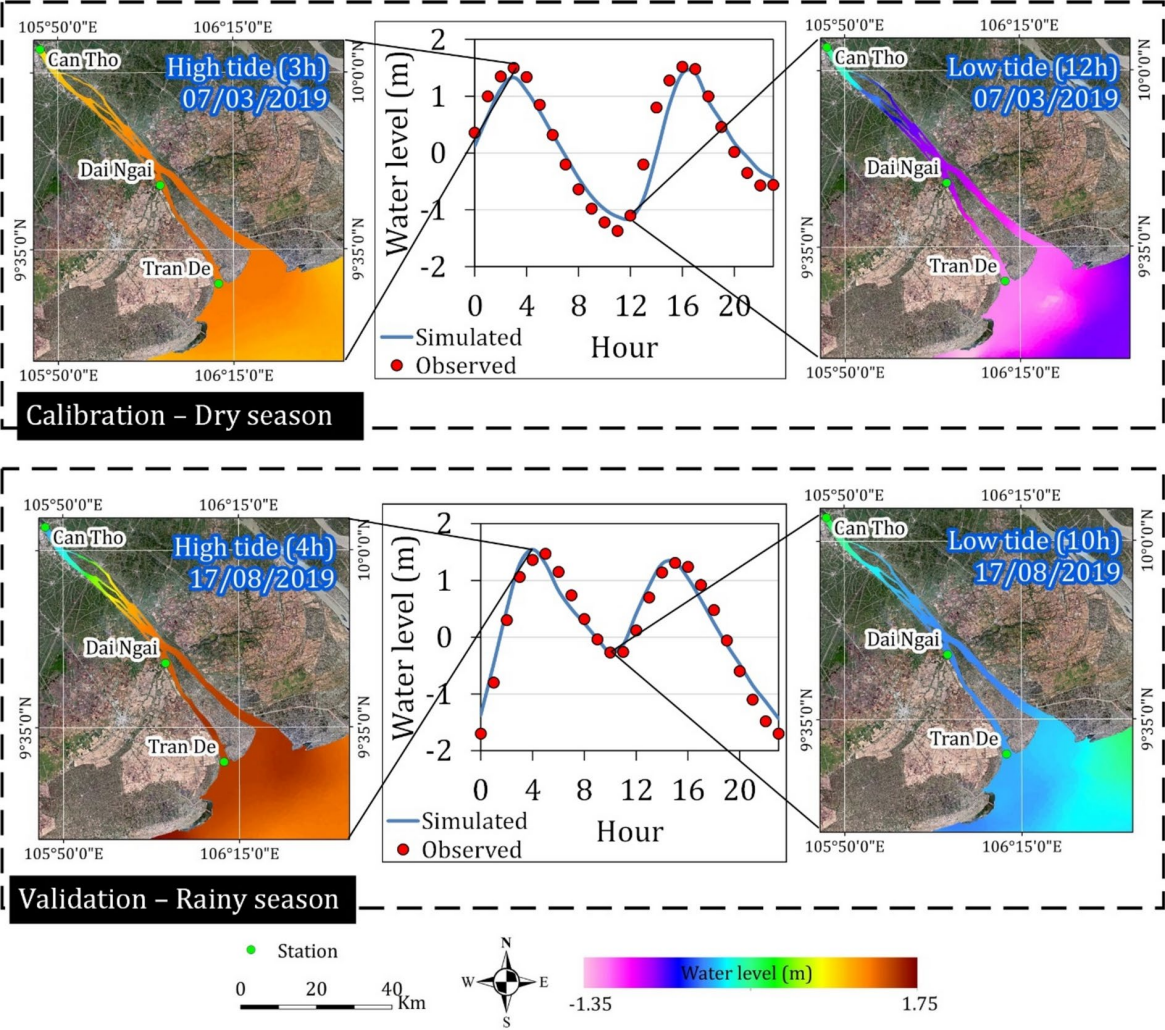
Illustrations of daily water level peaks and troughs during dry season (observed data used for model calibration), and rainy season (observed data used for model validation). Maps were generated by QGIS 3.16.0 (https://qgis.org).

Daily maximum and minimum water levels, commonly referred to as high and low tides, were identified and used to characterize seasonal water level patterns within the study area, as exemplified in Fig. 4. Typically, two tidal cycles occur daily with a time difference of approximately 1–2 h between seasons. Water level fluctuations exhibited seasonal variability, ranging from -1.35 m to 1.40 m during the dry season and expanding to 0.1 m to 1.75 m during the rainy months.

**Fig. 5:**
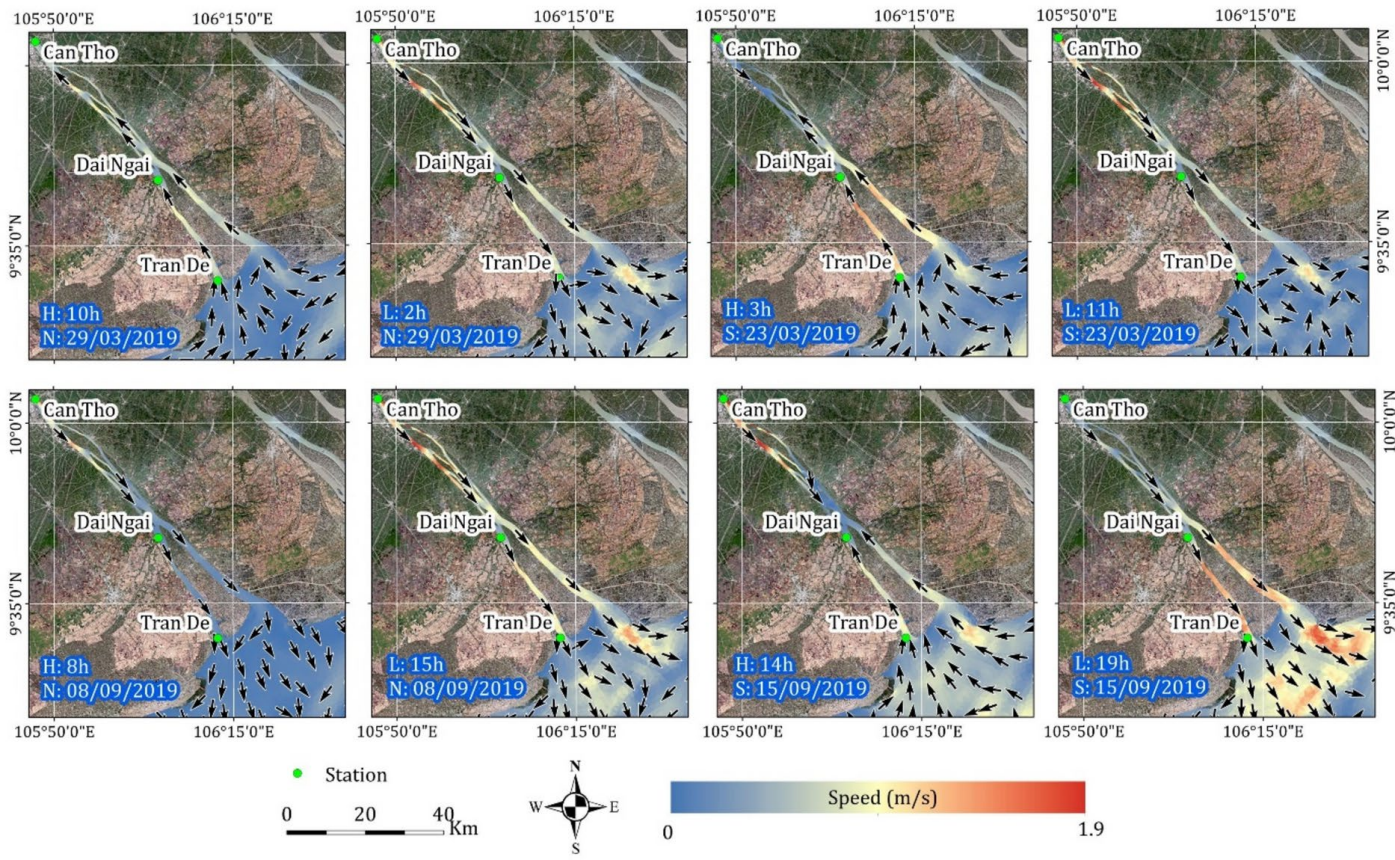
A comparison of speed and direction of water flow during dry (top) and rainy (bottom) seasons. H and L represent high tide and low tide during the day. S and T represent spring tide and neap tide during the month. Maps were generated by QGIS 3.16.0 (https://qgis.org).

The average flow velocities and associated directions measured in dry and rainy seasons reveal the reversal changes (Fig. 5). Water velocities are typically higher during rainy seasons due to increased upstream discharge associated with elevated precipitation levels. However, the direction of water flow exhibits a bi-directional pattern, influenced by tidal conditions. During dry seasons, water flow primarily adheres to physical forces, with seaward currents prevailing during low tides and landward currents during high tides, regardless of spring or neap tide phases. Conversely, the combination of high discharge and precipitation during rainy seasons generally drives a predominantly seaward flow. Nevertheless, exceptional instances arise during spring tides, such as on September 15, 2019, at 14:00, where the tide’s influence temporarily reverses the flow direction, leading to a landward current. The lunar tidal cycle, characterized by distinct variations between spring and neap tides, occurs monthly. Spring tides occur when the Earth, sun, and moon are aligned, while neap tides arise when the sun and moon are perpendicular. Typically, the cycle difference between spring and neap tides is seven days, resulting in two occurrences of each tide type per month.

**Fig. 6:**
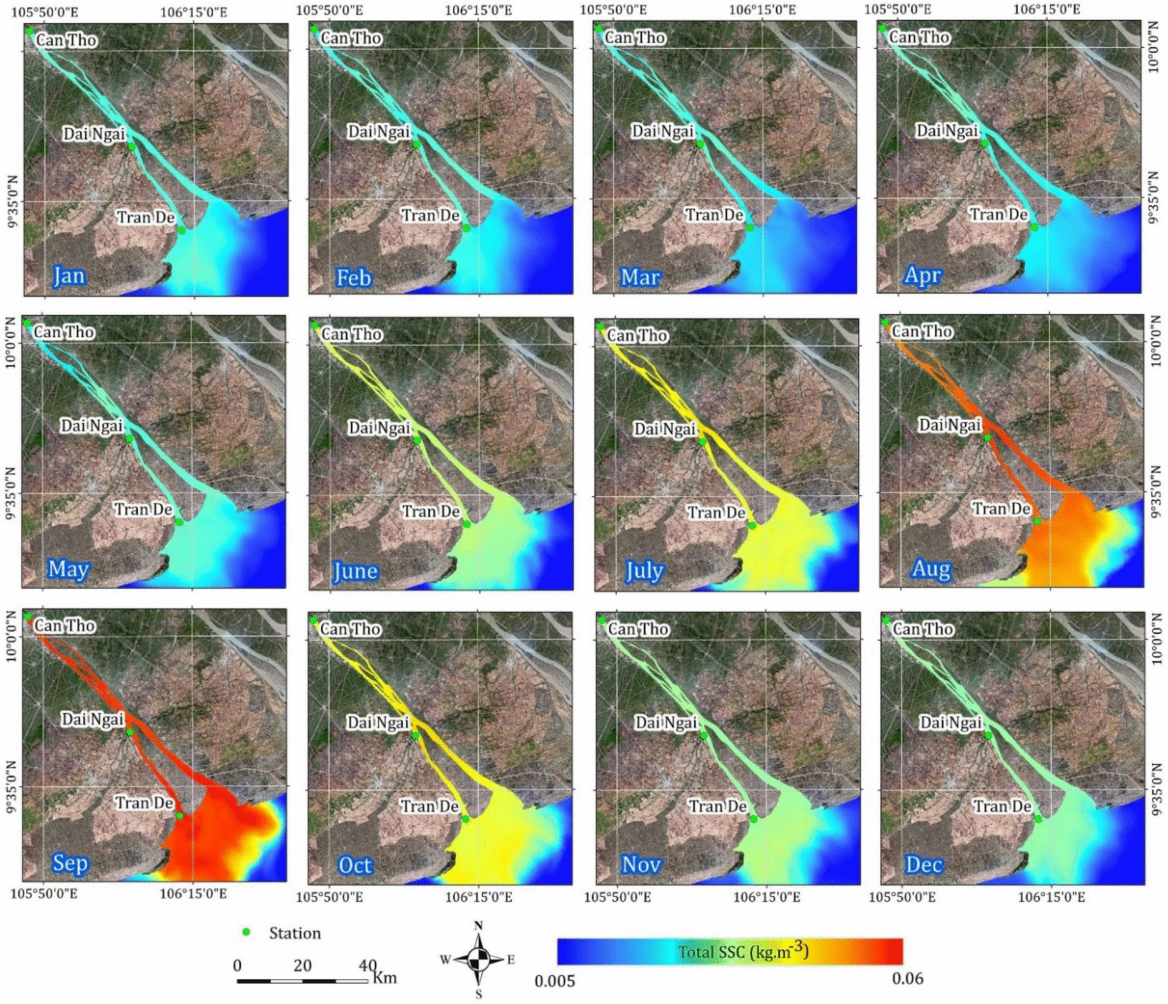
Typical monthly SSC calculated for the period 2019–2023. Maps were generated by QGIS 3.16.0 (https://qgis.org).

To characterize the spatial and temporal variability of SSC, mean monthly maps were generated spanning January 2019 to December 2023 (Fig. 6). The spatial distribution of SSC closely reflected natural hydrological conditions within the catchment, with elevated concentrations observed during the wet season coinciding with the annual flood pulse originating from the upper Mekong Delta. SSC exhibited in higher levels within the densely narrow network of inland river channels, and gradually attenuated downstream towards the coastal estuary. A seasonal cycle was evident, with minimum SSC occurring in April and a gradual increase culminating in a peak in September.

**Fig. 7:**
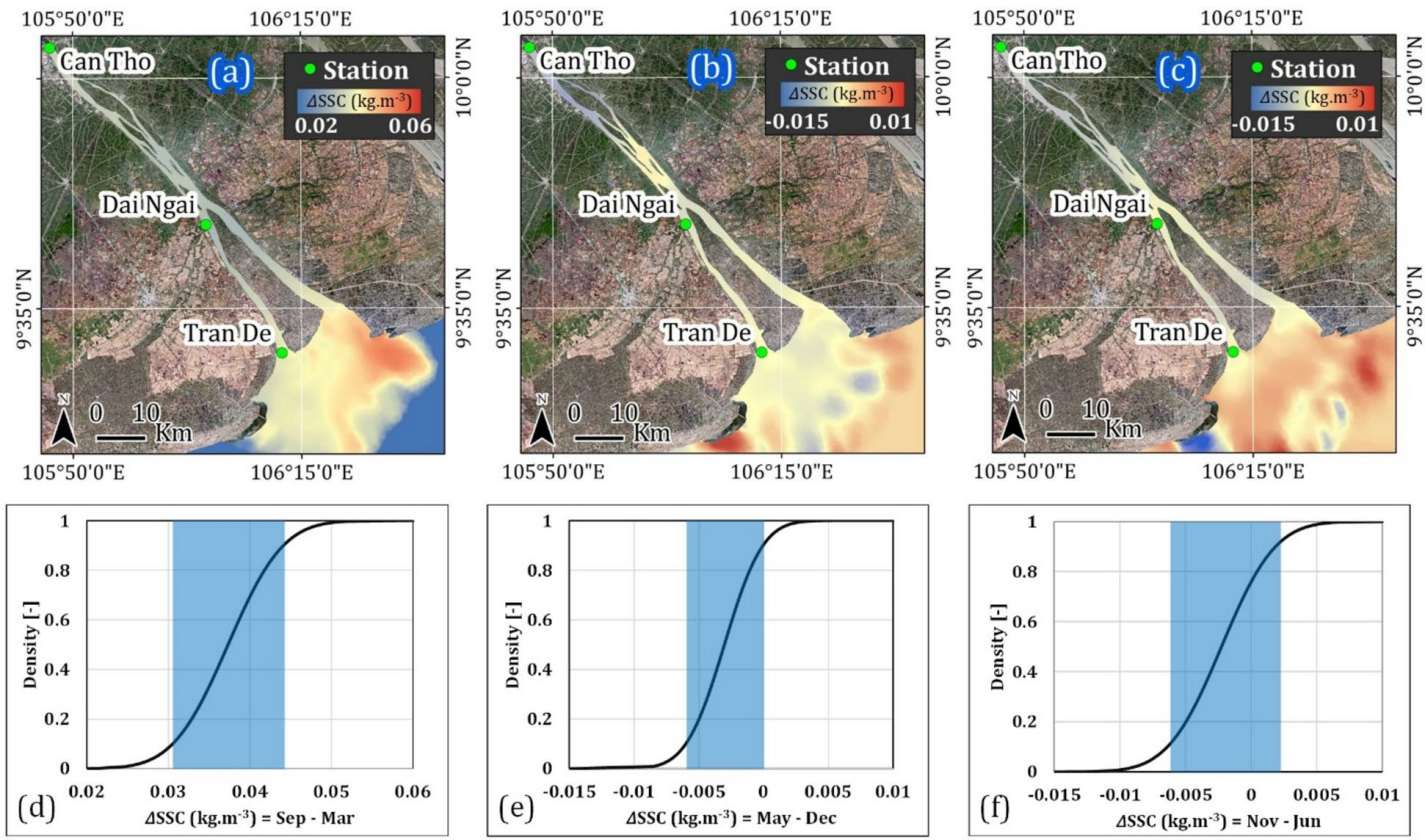
Typical spatial patterns (top) and corresponding cumulative distribution (bottom) represent SSC differences across specific periods: (**a**, **d**) during mid-rainy and mid-dry seasons (Sep–Mar); (**b**, **e**) the early and late dry seasons (May–Dec); (**c**, **f**) the early and late rainy seasons (Nov–Jan). The blue box indicates approximately 80% of pixel density. Maps were generated by QGIS 3.16.0 (https://qgis.org).

Typical spatial variations accompanied by statistical differences (leveraging cumulative distribution function) highlight the specific SSC changing level across various periods calculated average throughout the examined years, as illustrated in Fig. 7. The statistical significance analysed by one-way analysis of variance (ANOVA) resulted in p-values consistently less than 0.00001 in all compared periods, indicating strong statistical evidence supports the rejection of the null hypothesis. A meaningful analysis of pixel density distribution indicates that the majority (80%) of pixels fall within the SSC range of 0.03 to 0.044 kg m^−3^ during the mid-rainy and mid-dry seasons (September-March). In contrast, pixel densities from the beginning to the end of dry seasons (May-December) and rainy seasons (November-June) are concentrated within narrower ranges, ranging from − 0.006 to 0 kg m^−3^ and − 0.005 to 0.002 kg m^−3^, respectively.

**Fig. 8:**
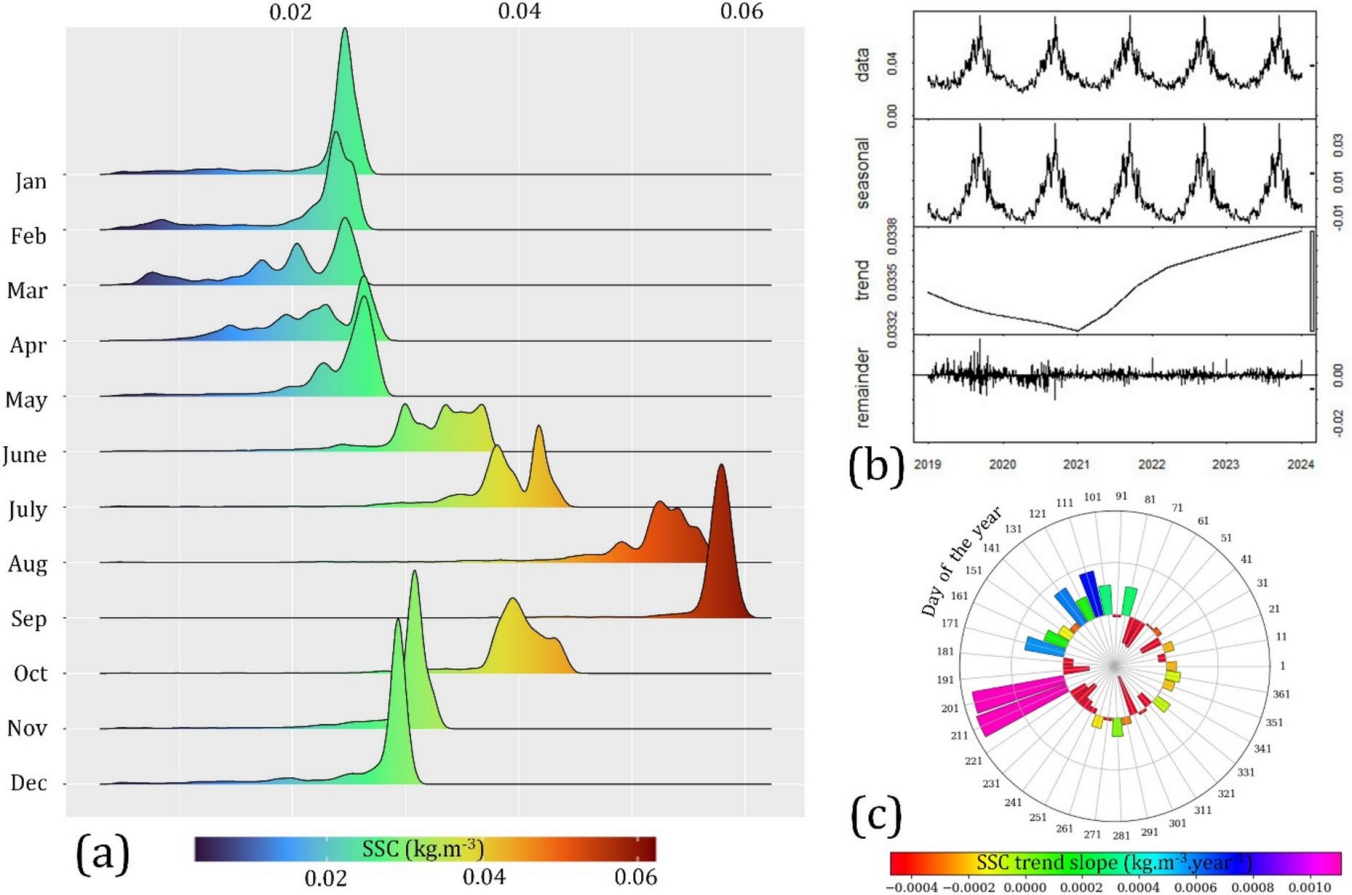
(**a**) Histogram density distribution of monthly SSC, (**b**) time series decomposition, and (**c**) 10-day trend slope.

The ridgeline plot illustrates a clear peak in SSC during the mid-rainy period of September, followed by a significant decline to the lowest levels in April of the dry season, as depicted in Fig. 8a. September exhibited a quasi-normal distribution of SSC, with a mean value of approximately 0.057 kg m^−3^. In contrast, the dry months demonstrated a wider dispersion of SSC values compared to the rainy period. The lowest mean SSC occurred in April, which was only three times lower than the September mean.

Due to the significant influence of the tropical monsoon climate on hydrological flow, the seasonal amplitude during dry and rainy periods contributed significantly to the time series observations. Hence, the locally estimated scatterplot smoothing (LOESS) method was applied to decompose the simulated daily SSC time series (2019–2023) into three distinct components: trend, seasonal amplitude, and remainder (Fig. 8b). The analysis revealed a range of seasonal amplitudes from -0.013 to 0.042 kg m^−3^. Moreover, the remainder component, representing anomalies, was consistently higher during rainy seasons (0.01–0.015 kg m^−3^) compared to dry seasons (0.004–0.007 kg m^−3^). Upon excluding the dominant seasonal variability, two distinct trends emerged: a decrease during the sub-period 2019–2020 and a subsequent increase during 2021–2023. Note that, the upward trend 2021–2023 is likely to correspond with La Nina (https://origin.cpc.ncep.noaa.gov), also known as the cold phase of El Niño-Southern Oscillation (ENSO) as an increase in precipitation and discharge over coastal Mekong Delta^33^.

In order to acquire a comprehensive understanding of seasonal trends, daily data were aggregated into 10-day averages, resulting in 37 observations per year. These 10-day averages from the past five years (2019–2023) were subjected to linear regression analysis, with the resulting slopes depicted in Fig. 8c. The polar chart reveals a notable upward trend concentrated within days 201 to 211, coinciding with the early rainy season in July. During this period, the rate of change was approximately 0.0010 kg m^−3^. Moreover, a pronounced upward trend was observed over the past five years, particularly during the late dry season. Conversely, a slight downward trend was typically evident at the beginning of both dry and rainy season.

## Discussion

This study utilized a robust hydrodynamic modelling framework to investigate the intricate dynamics of fluvial-marine continuum flow. This requires a rigorous construction of a hydrological model by incorporating hourly station data from inland fluvial (CT and DN stations) and estuarine entrances (TD stations) across various climatological periods. By strategically selecting calibration data from dry months and validation during rainy months, the model effectively captured the diverse flow regimes, enabling accurate model adjustment for long-term time series prediction. Compared to previous studies focusing on specific seasons or sub-periods^34–37^, our model achieved comparable performance in simulating complex hydrological runoff within the downstream Mekong Delta, while accurately capturing the dynamics of SSC using daily time series analysis over 5 recent years.

A pronounced seasonal oscillation in SSC was observed, with values gradually increasing from the dry season to the rainy season (Figs. 6 and 8a). This is consistent with the gauging station datasets used for calibration and validation, which demonstrated a substantial difference in SSC between these two distinct periods. The tropical monsoon climate, characterized by high-frequency precipitation and significant upstream water discharge during rainy seasons, contributes to higher flow rates compared to dry seasons. Previous studies have highlighted the substantial influence of tropical monsoon seasons within the study area^35,38^. Moreover, seasonal hydrological fluctuations driven by tidal mechanisms significantly impact sediment transport, nutrient cycling, floodplain vegetation, fish habitats, and fisheries in the Mekong River basin. Our results revealed significant changes in water level, flow velocity, and direction influenced by high tide, low tide, spring/neap tide phases, and dry/rainy seasons (Figs. 5 and 7). Ultimately, we emphasized the role of quantifying the annual seasonal amplitude to capture precise trends through time series decomposition (Fig. 8b).

We mainly based on a numerical model and limit the range of SSC dynamics in 5 recent years (2019–2023) based on the availability of input parameters. For long-term decadal monitoring, future efforts should incorporate data assimilation techniques that leverage both numerical modelling and remote sensing data. Numerical models, mathematical representations of physical systems, are widely utilized to simulate water flow and related processes^26,27^. Conversely, remote sensing acquires objective information pertaining to water surface characteristics^39,40^. Challenges in numerical models necessitate a comprehensive dataset of accurate input parameters, including bathymetry and boundary conditions, along with meticulous calibration to capture the full spectrum of physical hydrodynamics and uncertainties. In contrast, remote sensing can be constrained by atmospheric conditions, sensor capabilities, and data processing challenges. While numerical models provide detailed information about water flow patterns, remote sensing data can serve as valuable initial conditions for model configuration and intercomparison. However, significant challenges remain in data assimilation of these two approaches, particularly due to spatial mismatches between the unstructured triangular mesh of numerical models and pixel-based remote sensing data. This challenge is exacerbated by cloud contamination, which hinders optical satellite observations, especially in tropical monsoon climates^41^. Overcoming these technical limitations is essential to establish a long-term dataset and deepen our understanding of sediment dynamics in the complex coastal Mekong River.

Along the Mekong river-estuarine continuum, our findings revealed that significant variations in flow velocity and direction induce fluctuations in water level and sediment budget periodically. During the rainy season, increased SSC is attributed to the dominance of fluvial inputs following high-discharge periods associated with the monsoon, which transports substantial quantities of sand and mud downstream^12,42^. Tidal modulation, including flow reversals and bed shear stress, further influences sediment transport by redistributing and mixing materials along the river-estuarine continuum^42,43^. Conversely, during low-discharge seasons, estuarine circulation promotes sediment trapping and mud deposition, reducing bed material mobility and leading to decreased sediment transport dynamics^12,42,43^. The intricate sediment dynamics of the coastal Mekong River are shaped by a complex interplay of fluvial, tidal, and marine processes, exhibiting distinct seasonal patterns. Consequently, both anthropogenic activities, such as upstream damming^44^, and sea-level rise^45^ pose cumulative threats, altering sediment supply and influencing coastal morphodynamics. These changes underscore the urgent need for ongoing monitoring to mitigate potential adverse impacts.

Regarding model structure, we employed a 2D hydrodynamic model, a widely recognized standard for simulating sediment transport in local river-estuarine systems^37,46,47^. We demonstrated the rigorous calibration and verification across various temporal and spatial scales, resulting in reliable 5-year time-series simulations. While 3D models offer the potential for more accurate representation of vertical flow structures and sediment density, their application is recommended for specific cases such as hyperpycnal flows and short-term morphological responses^46^. Moreover, long-term simulations with 3D models can be computationally demanding^47^. For complex coastal regions like the Mekong Delta, a 3D model offers a promising solution, yet requires exhaustive input data on vertical properties during both low-flow dry seasons and high-flow rainy periods, along with upstream discharge and detailed physical coastal properties.

A detailed analysis of 10-day observations over the past five years reveals a distinct upward trend in sediment transport during low-flow dry seasons, while downward trends were observed predominantly during high-flow rainy periods (Fig. 8c). This highlights the specific periods of sediment budget within the downstream Mekong coastal region. Over the last decades, both natural conditions (ocean dynamics, wave, tidal)^48^ and human interference (dam building, sand mining)^44^ were assessed related to the decrease of sediment over the sensitive coastal region. The transboundary nature of the Mekong Basin, encompassing six countries (China, Laos, Thailand, Myanmar, Cambodia, and Vietnam)^49^, underscores the need for synchronous management and coordination among stakeholders. While basin-wide efforts to fully restore seasonal hydrological variability may be challenging within the current institutional framework, targeted re-operation strategies in the Lower Mekong region could offer viable opportunities for partially recovering key hydrological characteristics without compromising stakeholder coordination^50^. In this context, identifying specific periods of abundant sediment is crucial in supporting nature-based solutions for coastal restoration and sustainable use^51^.

## Materials and methods

### Study area

**Fig. 9:**
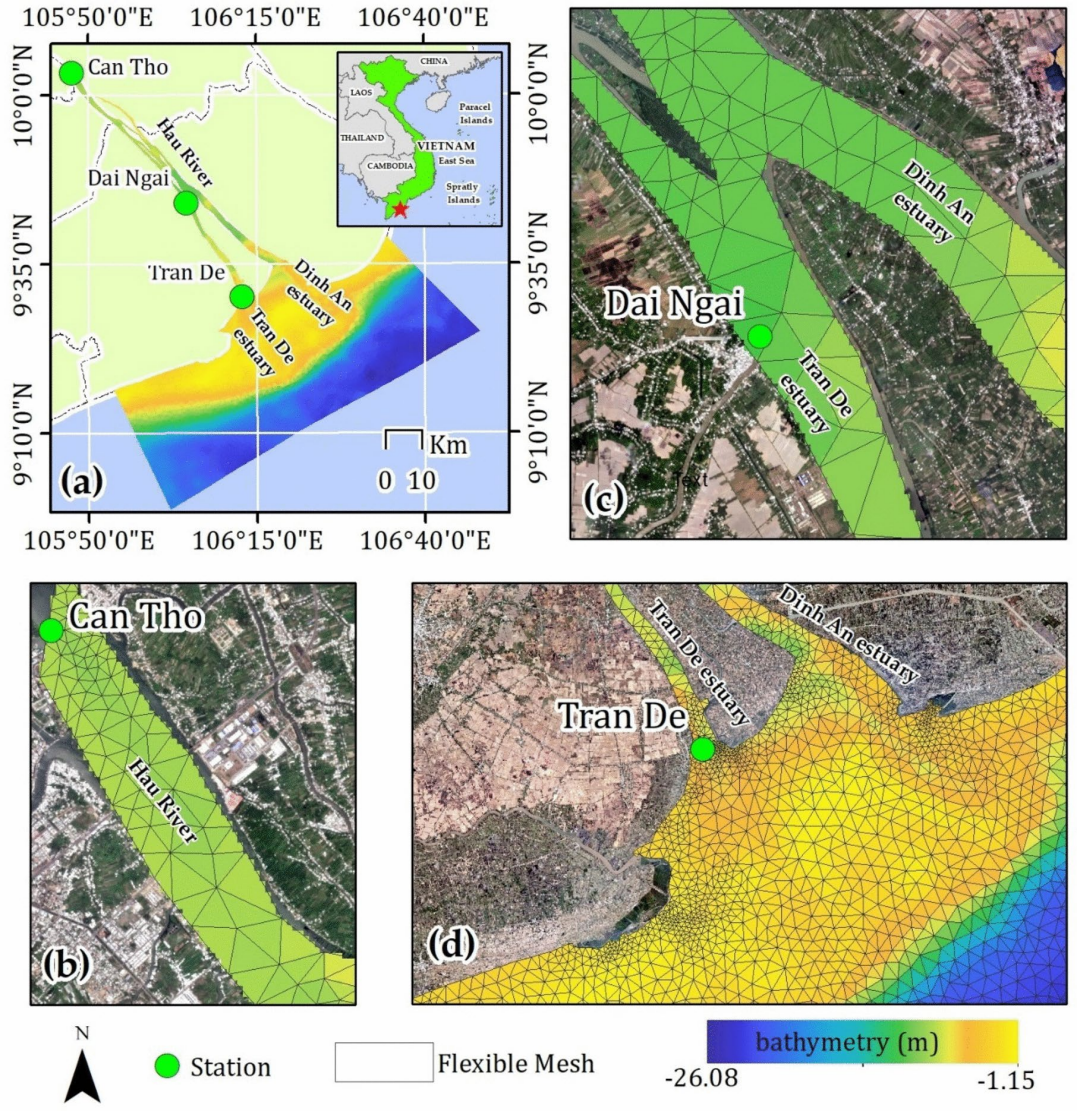
Map showing the location of the study area (**a**) with zoom in different areas following bathymetry background and flexible mesh: (**b**) riverine, (**c**) transitional zone, and (**d**) coastal zone. Maps were generated by QGIS 3.16.0 (https://qgis.org).

Our focus was on the Hau River, a major tributary of the lower Mekong River (Fig. 9). Situated within the coastal floodplain, the region experiences a tropical monsoon climate characterized by abundant precipitation and annual flooding during the rainy season. This climatic regime is intimately linked to the interplay between flow conditions and sediment dynamics. The dry season typically spans from December to May, transitioning to the rainy season from June to November. As previously mentioned, the river separates prior to reaching the sea, forming the Tran De and Dinh An estuary. Dinh An estuary exhibits a concentrated flow, accounting for 70% of the total flow during the dry season and exceeding 90% during the rainy season, predominantly influenced by ebb tides^37^. In contrast, Tran De estuary is characterized by a predominance of flood tides^37^. Following flow seaward, bathymetry exhibits a range from a shallowest of 1.15 m to a maximum depth of 26.07 m.

### Datasets

Observed data were collected at various locations and periods spanning the river channel to the coastal estuary, from stations operated by the Southern Hydro-meteorological Station under the Vietnam Ministry of Natural Resources and Environment: Can Tho (CT), Tran De (TD), and Dai Ngai (DN). Hourly discharge data were collected at the CT station, while SSC samples were obtained at all three years (2018, 2019, 2021) and analysed in a laboratory using the filtration method. A sample was passed through a filter paper, dried at 103–105 °C for one hour, and then weighed to determine SSC. Water level data were recorded hourly at TD and DN stations during both dry (February, March, and April) and rainy seasons (August, September, and October) in 2019. This dataset was essential for capturing the complex hydrodynamic variations associated with the tropical monsoon climate. Bathymetric data for the study area were acquired using a multi-beam current gauge and an acoustic Doppler current profiler (Teledyne ADCP—RDI Workhorse 600 kHz) mounted on a boat. This equipment was used to measure cross-sectional and longitudinal profiles of the river channel. Tidal predictions in the MIKE21 model are generated by assimilating tidal gauge records of the primary tidal constituents^52,53^. These assimilated data are then used to forecast water levels at open seaward boundaries. Details of datasets are provided in Table 1.

**Table 1:** Statistics of datasets used in the present study.

Datasets	Station	Time	Unit	Max	Min	Note
Water level	Tran De	(February to April; August to October) 2019	m	2.36	− 1.9	Hourly
Water level	Dai Ngai	(February to April; August to October) 2019	m	2.15	− 1.81	Hourly
Water level	Sea	2019	m	1.75	− 2.89	Hourly
Discharge	Can Tho	2019	m^3^/s	23,700	0	Hourly
SSC	Can Tho	2018; 2019; 2021	kg/m^3^	0.201	0.0081	Daily
Bathymetry	Hau river	2016–2020	m	− 1.15	− 26.08	

### Numerical hydrological model construction

This study employed the MIKE 21 modeling system, integrating the hydrodynamic module (HD) with the sediment transport module (MT), to analyze the spatiotemporal variations of hydrological characteristics and SSC within a complex fluvial-marine continuum. The MIKE 21 system, developed by the Danish Hydraulic Institute, is a two-dimensional (2D) numerical model grounded in shallow water and integrated Navier–Stokes equations. Its ability to create unstructured triangular meshes provides flexibility in addressing complex shallow water domains, making it a prevalent tool in hydrological research^54–56^.

#### Theoretical basis

The simulation of flow and water level variations is based on the continuity and momentum equations in two directions^57^. For continuous equation:

1 $$\frac{\partial\text{h}}{\partial\text{t}}+ \frac{\partial\text{h} - {\text{u}}}{\partial\text{x}}+ \frac{\partial\text{h} - {\text{v}}}{\partial\text{y}}= \text{hS}$$

Momentum equation in the x direction:

2 $$\frac{{\partial {\text{hu}}}}{{\partial {\text{t}}}} + \frac{{\partial {\text{hu}}^{{\text{2}}} }}{{\partial {\text{x}}}} + \frac{{\partial {\text{huv}}}}{{\partial {\text{y}}}} = {\text{fvh}} - {\text{gh}}\frac{{\partial \eta }}{{\partial {\text{x}}}} - \frac{{\text{h}}}{{\rho _{0} }}\frac{{\partial {\text{p}}_{{\text{a}}} }}{{\partial {\text{x}}}} - \frac{{{\text{gh}}^{2} \partial \rho }}{{2\rho _{0} \partial {\text{x}}}} + \frac{{\tau _{{{\text{sx}}}} }}{{\rho _{0} }} - \frac{{\tau _{{{\text{bx}}}} }}{{\rho _{0} }} + \frac{\partial }{{\partial {\text{x}}}}\left( {{\text{hT}}_{{{\text{xx}}}} } \right) + \frac{\partial }{{\partial {\text{y}}}}\left( {{\text{hT}}_{{{\text{xy}}}} } \right) + {\text{hu}}_{{\text{s}}} {\text{S}}$$

Momentum equation in the y direction:

3 $$\frac{\partial\text{h} - {\text{v}}}{\partial\text{t}}+ \frac{\partial\text{h} - {\text{uv}}}{\partial\text{x}}+ \frac{\partial\text{h}{ - {\text{v}}}^{2}}{\partial\text{y}}= \text{f} - {\text{u}}\text{h-gh}\frac{\partial \eta}{\partial \text{y}} - \frac{\text{h}}{{\rho}_{0}} \, \frac{\partial {\text{p}}_{\text{a}}}\partial{\text{y}}- \frac{{\text{g}}{\text{h}}^{2}}{{2}{{\rho}}_{0}}\frac{\partial \rho}{\partial\text{y}}+ \frac{{\tau}_{\text{sy}}}{{\rho}_{0}} - \frac{{\tau}_{\text{by}}}{{\rho}_{0}}+ \frac{\partial}{\partial \text{x}}\left({\text{h}}{\text{T}}_{\text{xy}}\right)+ \frac{\partial}{\partial\text{y}}\left({\text{h}}{\text{T}}_{\text{yy}}\right)+ {\text{hv}}_{\text{s}}{\text{S}}$$

where, t is the time variable; x, y are Cartesian co-ordinates on the horizontal plane; h is water depth (m), S is the magnitude of the discharge due to point source (m^3^/m/m^2^); f is Coriolis parameter (rad/s); g is gravitational acceleration (m/s^2^); p_0_ is reference density of water (kg/m^3^); p is density of water (kg/m^3^); p_a_ is atmospheric pressure (N/m^2^); u_s_, v_s_ are the velocity by which the water is discharged into the ambient water (m/s); T_sx_, T_sy_ are surface friction stress in two directions x, y (N/m^2^); T_bx_, T_by_ are bottom friction stress in two directions x, y (N/m^2^);

The sediment transport module is established based on the transport–diffusion equation^58^:

4 $$\frac{\partial \overline{c}}{\partial t }+u\frac{\partial \overline{c}}{\partial x }+ v\frac{\partial \overline{c}}{\partial y }= \frac{1}{h} \frac{\partial }{\partial x} \left(h{D}_{x} \frac{\partial \overline{c}}{\partial x }\right)+ \frac{1}{h} \frac{\partial }{\partial y} \left(h{D}_{y} \frac{\partial \overline{c}}{\partial y }\right)+ {Q}_{L}{C}_{L}\frac{1}{h}+\frac{1}{h}-S$$

where, t is the time variable; x, y are Cartesian co-ordinates on the horizontal plane; $\overline{\text{c} }$ is depth average concentration (g/m^3^); Dx, Dy are diffusion coefficient in x, y directions (m^2^/s); S is deposition/erosion term (g/m^3^/s); Q_L_ is source discharge per unit area (m^3^/s/m^2^); C_L_ is concentration of the source discharge (g/m^3^); h is water depth (m); u, v are depth average flow velocities (m/s).

#### Calculation grid and parameter setting

To represent accurately the complex hydrodynamic processes within the study area, which encompasses a river system extending from the inland to the coastal water, an unstructured triangular grid methodology was employed. The domain was divided basically into three distinct zones: riverine, transitional, and coastal. To capture the intricacies of bathymetry and riverbank geometries in critical areas while maintaining computational efficiency, a variable grid resolution was implemented. Edge sizes were set at 100 m in the riverine zone, 500–300 m in the transitional zone, and 1000 m in the coastal zone (Fig. 9). The resulting mesh, consisting of 3536 nodes and 6243 elements, following to a quality standard with all triangular angles less than 26°. After calculating the mesh and setting up the simulation for the model, the parameters are listed in Table 2.

**Table 2:** Model parameterization.

Parameter	Value	Unit
Water level module		
Eddy viscosity	0.28	m^2^/s
Manning coefficient	55–120	m^1/3^/s
Time step	3600	s
Drying depth	0.005	m
Flooding depth	0.05	m
Wetting depth	0.1	m
Coriolis forcing	Varying in domain	deg
Velocity in x, y direction	0	m/s
Sediment module		
Density of sediment	2650	kg/m^3^
Concentration for flocculation	1	kg/m^3^
Concentration for hindered settling	5	kg/m^3^
Constant settling velocity coefficient	0.0009	m/s
The critical shear stress for deposition	0.3	N/m^2^
Erosion coefficient	1 × 10^–8^	kg/m^2^/s
The critical shear stress for erosion	0.5	N/m^2^
Bed roughness	1 × 10^–5^	m
Dispersion coefficient formulation	1	m^2^/s

#### Boundary conditions

Boundary conditions provide crucial information related to the inputs and outputs of the model, ensuring that the simulated results are realistic and representative of the real-world system. Therefore, limitations of the boundaries in the present study were configured based on the positions of observed stations. The model’s upstream boundary was set up at CT station as inputs of discharge and SSC from Hau River. For downstream, open boundaries were defined toward the sea as representing water level fluctuations within the coastal waters. An open boundary condition with an average radius of approximately 35 km from TD station was established on the offshore boundary. This minimizes the influence of open ocean conditions on the simulated flow and wave fields within the estuarine domain. Water level elevations at this boundary were obtained from tidal predictions generated by MIKE 21, while SSC was set to 0 kg m^−3^.

## Evaluation strategies

The kernel density function was employed to conduct linear regression analysis and assess the model performance. Outlier removal was achieved by restricting the analysis to data points within the 5th and 95th percentiles of the confidence interval. Independent observed station datasets were used for both calibration and validation. For water level simulation, three months of dry season data (February, March, and April) from the TD and DN stations in 2019 were used for calibration, while the rainy season (August, September, and October) data were used for validation. For SSC prediction, daily observations from the CT stations in 2019 and 2021 were utilized for calibration and validation, respectively. The performance of the model was evaluated qualitatively using three widely recognized statistical metrics: the Pearson correlation coefficient (R^2^), the root mean square error (RMSE), and the mean absolute error (MAE).

5 $${R}^{2}={\left(\frac{\sum_{i=1}^{n}\left({y}^{i}- \overline{y }\right)\left({y}^{i}- \overline{y }\right)}{\sqrt{\sum_{i=1}^{N}\left({y}^{i}- \overline{y }\right)\sum_{i=1}^{n}\left({y}^{i}- \overline{y }\right)}}\right)}^{2}$$

6 $$\text{RMSE= }\sqrt{\frac{1}{{\text{N}}}{\sum_{\text{i=1}}^{\text{N}}{\text{(y}}_{\text{i}} - {\text{x}}_{\text{i}}\text{)}}^{2}}$$

7 $$\text{MAE= }\frac{1}{{\text{n}}}\sum_{\text{i=1}}^{\text{n}} | {\text{y}}_{\text{i}} - {\text{x}}_{\text{i}} |$$

where, N is the number of samples, y_i_ is the simulated values estimated from model, x_i_ is the values recorded in hydrological stations.

As water levels change significantly over different locations and times, we further applied the Taylor diagram for comprehensive comparisons of model performance following different periods as well as individual stations^59^.

## Supplementary Information

Supplementary Figures.

## Data Availability

The datasets used and analysed during the current study are available from the corresponding author on reasonable request.
