# Supplementary material for: Spatiotemporal dynamics of suspended sediment in coastal Mekong Delta: a hydrodynamic modelling approach under tropical monsoon climate
Source: Sci Rep. 2025 Feb 18;15:5851. doi: 10.1038/s41598-025-89111-z (PMC11836357; doi:10.1038/s41598-025-89111-z)
Supplement: Supplementary file 1 — Supplementary Figures. [file 41598_2025_89111_MOESM1_ESM.docx]

**Supplementary information**

**Spatiotemporal dynamics of suspended sediment in coastal Mekong Delta: A hydrodynamic modelling approach under tropical monsoon climate**

Nguyen Ngoc An^a^, Pham Viet Hong^b^, Nguyen An Binh^a*^, Giang Thi Phuong Thao^a^, Le Van Tinh^c^, Nguyen Cao Hanh^a^, Thai Thanh Tran^d^.

^a^ Ho Chi Minh City Institute of Resources Geography, Vietnam Academy of Science and Technology, Ho Chi Minh City, Vietnam

^b^ Institute of Marine Geology and Geophysics, Vietnam Academy of Science and Technology, Hanoi, Vietnam

^c^ Ho Chi Minh City University of Natural Resources and Environment, Ho Chi Minh City, Vietnam

^d^ Institute of Tropical Biology, Vietnam Academy of Science and Technology, Ho Chi Minh City, Vietnam

*Corresponding author: [nabinh@hcmig.vast.vn](mailto:nabinh@hcmig.vast.vn)


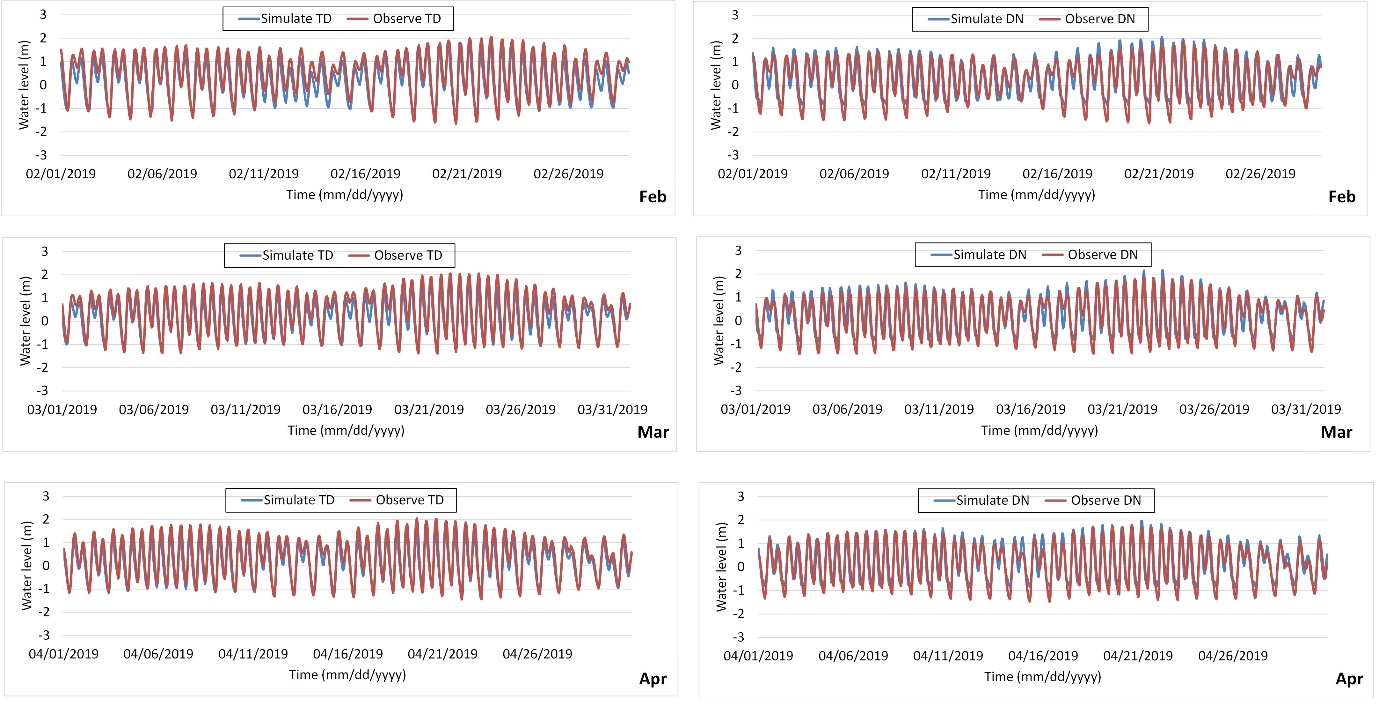


Figure S1. Time series of water levels used for calibration (February, March, and April) in Tran De (TD) station and Dai Ngai (DN) station. All data (without outlier removal) are included


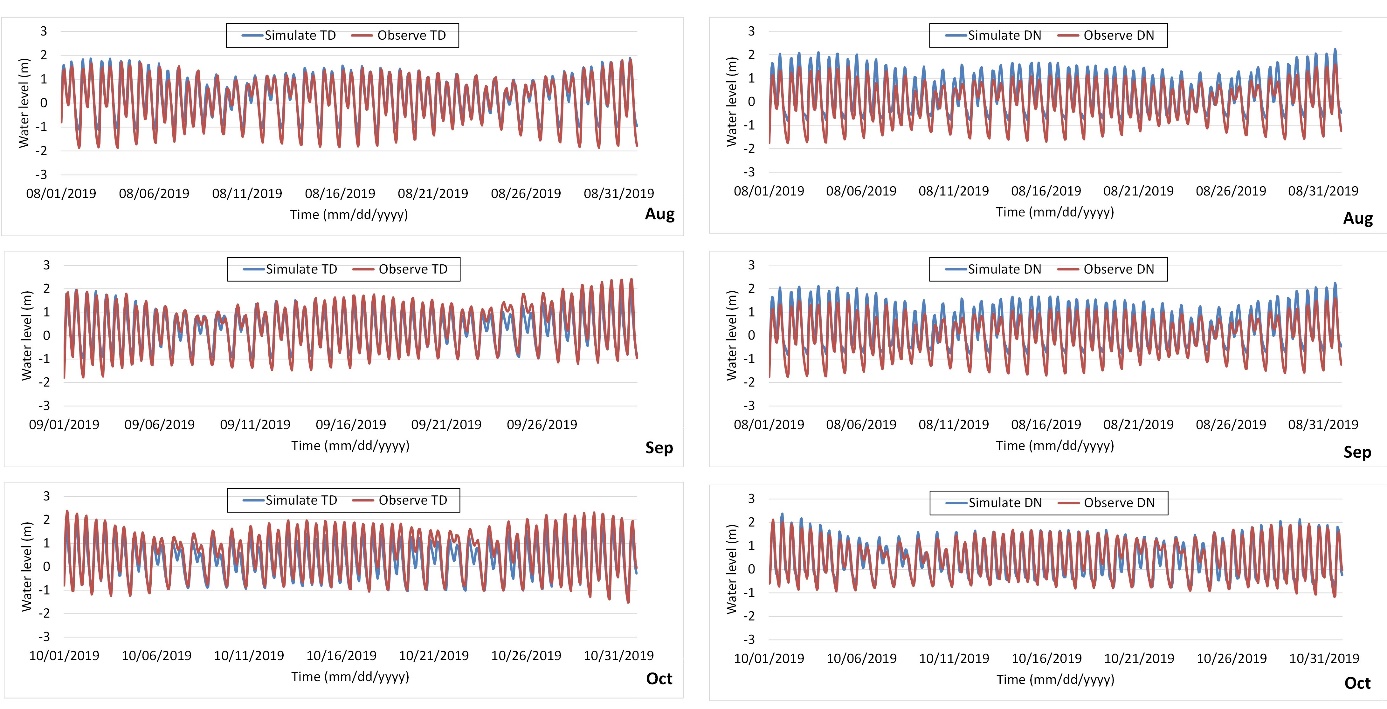


Figure S2. Time series of water levels used for validation (August, September, and October) in Tran De (TD) station and Dai Ngai (DN) station. All data (without outlier removal) are included


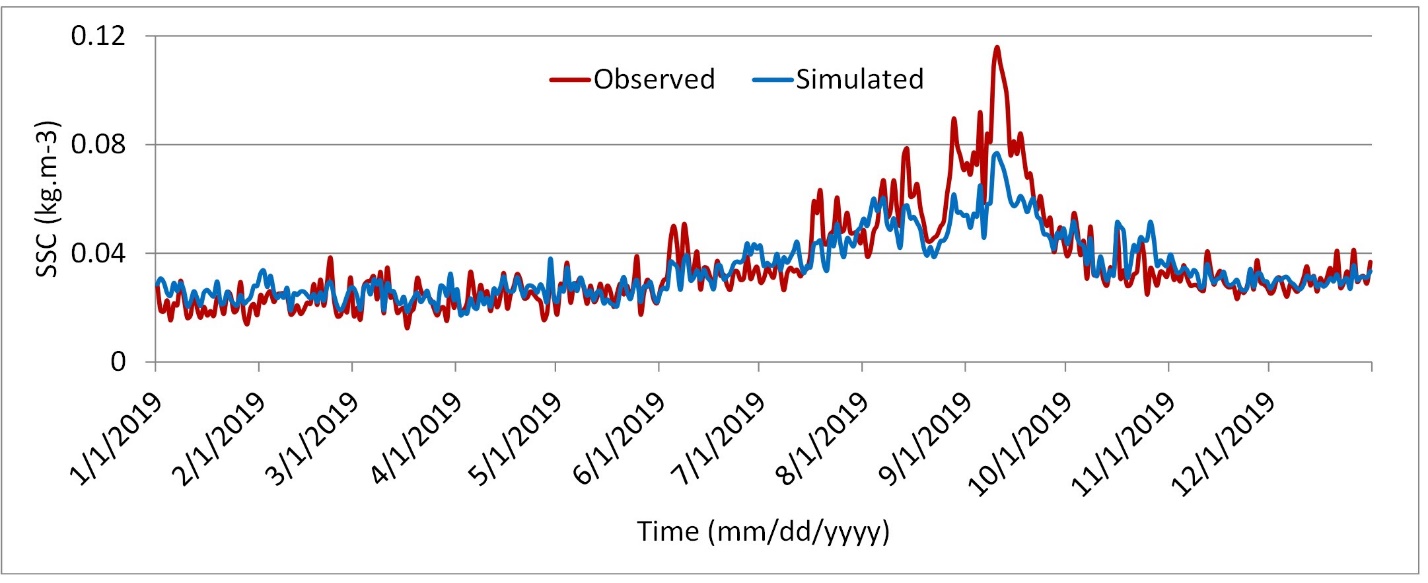


Figure S3. Time series of Suspended Sediment Concentration (SSC) used for calibration (2019) in Can Tho station. All data (without outlier removal) are included


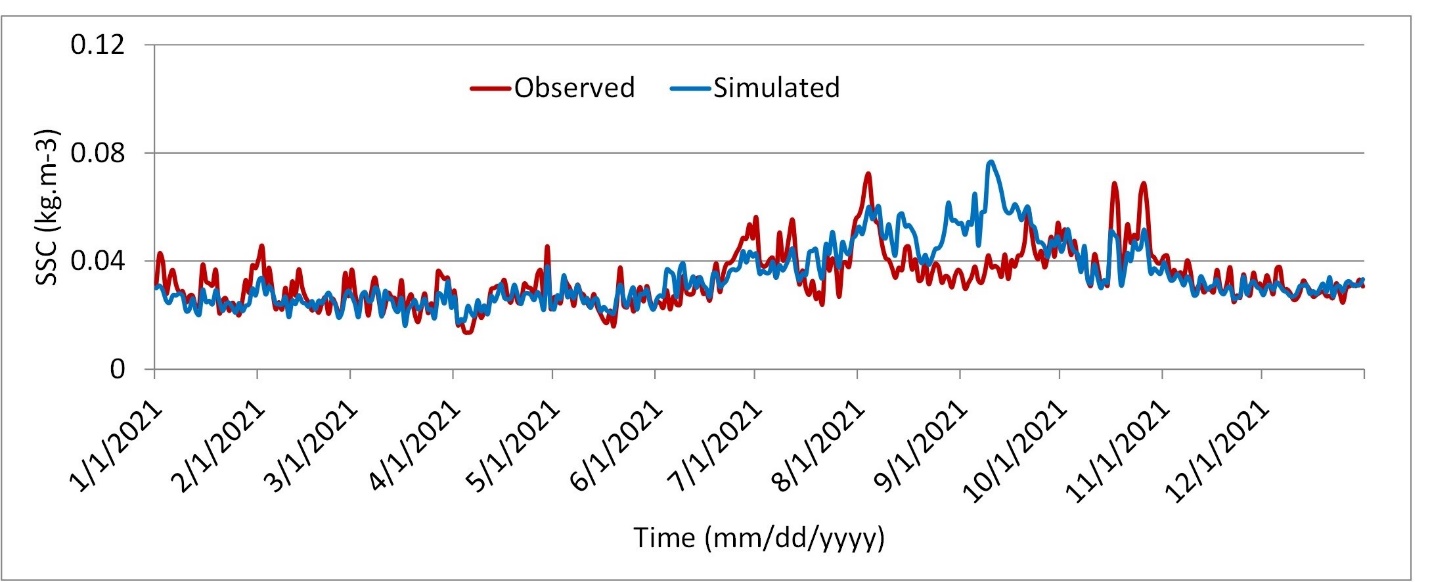


Figure S4. Time series of Suspended Sediment Concentration (SSC) used for validation (2021) in Can Tho station. All data (without outlier removal) are included
